# Placing a Biventricular Implantable Cardioverter Defibrillator in Major Cardiac Dextroposition (Pseudo-Dextrocardia): A Case Report and Literature Review

**DOI:** 10.7759/cureus.48454

**Published:** 2023-11-07

**Authors:** Abdelrahman S Abdalla, Chad Brands, Khawaja T Aziz, Thomas Shimshak, Denham Windross, Safi U Ahmed

**Affiliations:** 1 Department of Internal Medicine, AdventHealth, Sebring, USA; 2 Department of Cardiovascular Medicine, AdventHealth, Sebring, USA

**Keywords:** post-radiotherapy, pirfenidone, interventional cardiology, implantable-cardioverter defibrillator, pseudo dextrocardia

## Abstract

We present an unusual case of a geriatric patient with right-sided cardiac displacement and rotation (Pseudo-Dextrocardia) secondary to radiation-induced pulmonary fibrosis (RIPF) after radiation for carcinoma of the right breast. This patient with heart failure with reduced ejection fraction (HFrEF) underwent cardiac resynchronization therapy with a defibrillator (CRT-D) for primary prevention of sudden cardiac death. Cannulization of the coronary sinus ostium was difficult, likely due to the significant cardiac displacement. However, after multiple attempts, it was eventually successful. The clinical manifestations, evaluation, and technical and procedural issues in this patient with an unusual anatomic variant are summarized.

## Introduction

In its normal anatomical orientation, approximately one-third of the heart is situated to the right of the midsternal line, while the remaining two-thirds lie to the left. In cases of congenital dextrocardia, a developmental condition involving the transposition of the heart and other visceral organs, or sometimes the heart alone, the heart assumes a symmetrical position. In this state, roughly two-thirds of its mass is positioned to the right of the midsternal line, and one-third to the left, with the apex aligned along the right nipple line. Acquired displacement of the heart towards the right can range from barely noticeable to an extreme condition where the heart comes into contact with the lateral or posterolateral thoracic wall. Nevertheless, typically, the heart's long axis remains consistent, with the apex located farthest to the left [1].

Implantable cardioverter-defibrillators (ICDs) are a commonly employed intervention for preventing sudden cardiac death. While transvenous ICDs continue to be the primary form of treatment, there are certain constraints and associated complications. Additionally, careful consideration should be given to the placement of both the device and leads [2].

We reported a rare case of acquired cardiac displacement and rotation resulting from radiation-induced pulmonary fibrosis following treatment for right breast carcinoma. The patient underwent transvenous biventricular ICD implantation for the treatment of heart failure and primary prevention of sudden cardiac death.

## Case presentation

A 70-year-old female with a past cardiac history of class 3 congestive heart failure, non-ischemic dilated cardiomyopathy with left ventricular (LV) ejection fraction 20-25%, and left bundle branch block presented for outpatient implantation of a biventricular ICD for the treatment of heart failure and the primary prevention of sudden cardiac death. Past medical history was also notable for breast cancer, right-sided mastectomy, radiation therapy, and chemotherapy with adriamycin in the distant past along with hypothyroidism. Physical examination at the time of presentation was remarkable for diminished breath sounds in the right chest.

**Figure 1:**
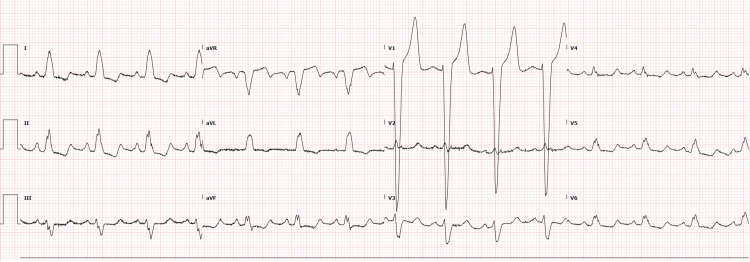
Initial ECG The electrocardiogram is showing sinus rhythm, normal PR-interval, and Left Bundle Branch Block (LBBB).

The 12-lead electrocardiogram demonstrated normal sinus rhythm, normal PR-interval, and Left Bundle Branch Block (LBBB) with QRS duration of 170 milliseconds as well as diffuse non-specific ST changes secondary to the LBBB (Figure 1). 

**Figure 2:**
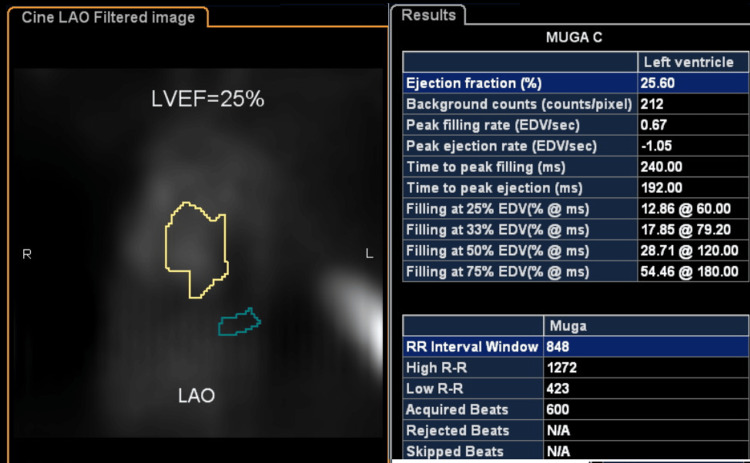
MUGA scan of the patient before ICD (CRT-D) implantation. MUGA: Multigated acquisition; ICD: Implantable cardioverter-defibrillators; CRT-D: Cardiac resynchronization therapy with a defibrillator.

A trans-thoracic echocardiogram revealed mildly dilated left ventricle, mild regurgitation in the mitral and tricuspid valves, ejection fraction of 20-25% with regional wall motion abnormalities, severe hypokinesis in the base to mid inferoseptum, base to mid anteroseptum, the base to mid inferior wall, and right ventricular filling pressure of 22 mm Hg. The multigated acquisition (MUGA) scan revealing a low left ventricular ejection fraction is shown in Figure 2.

**Figure 3:**
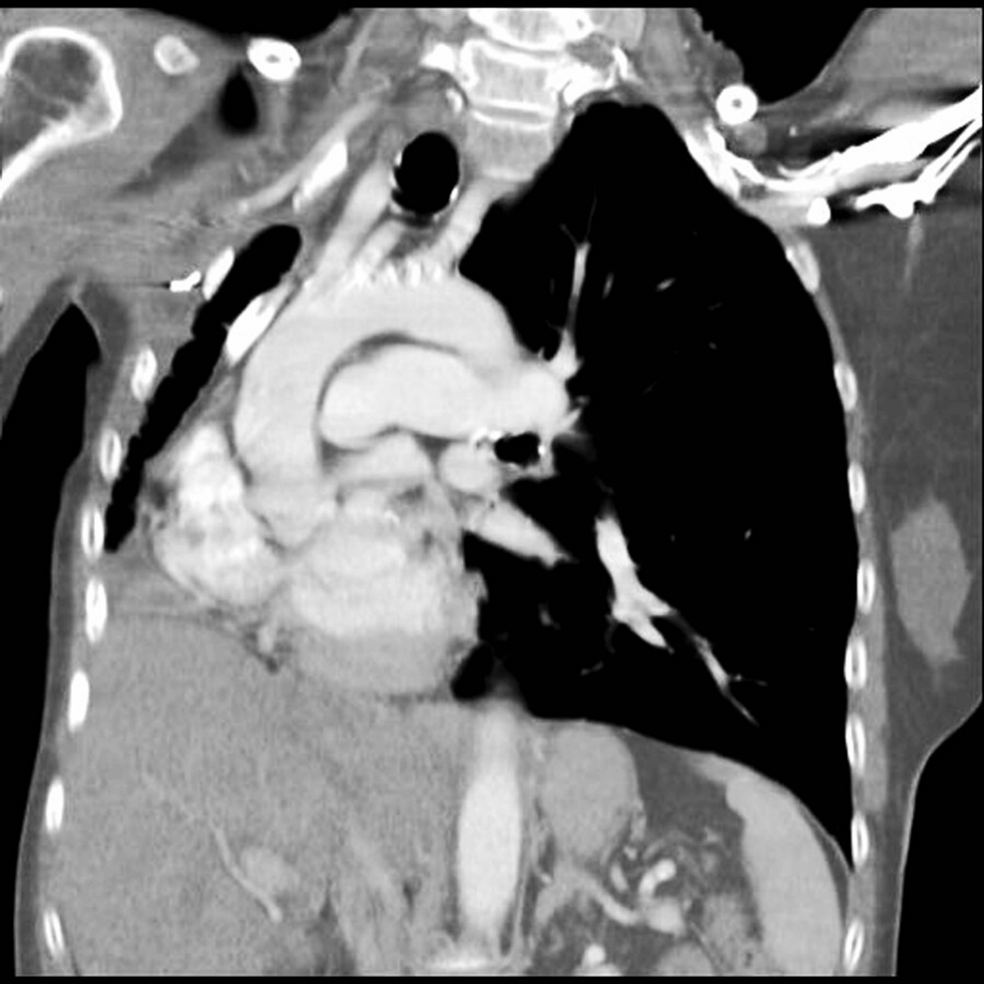
CT scan chest (pre-operative) Demonstrating marked volume loss of the right lung with bronchiectatic changes.

Computed tomography (CT) scan of the chest revealed marked volume loss of the right lung with diffuse bronchiectatic changes (Figure 3). This significant loss of lung volume and fibrosis led to the marked displacement of the heart to the right with intact baso-apical orientation.

Procedure summary

**Figure 4:**
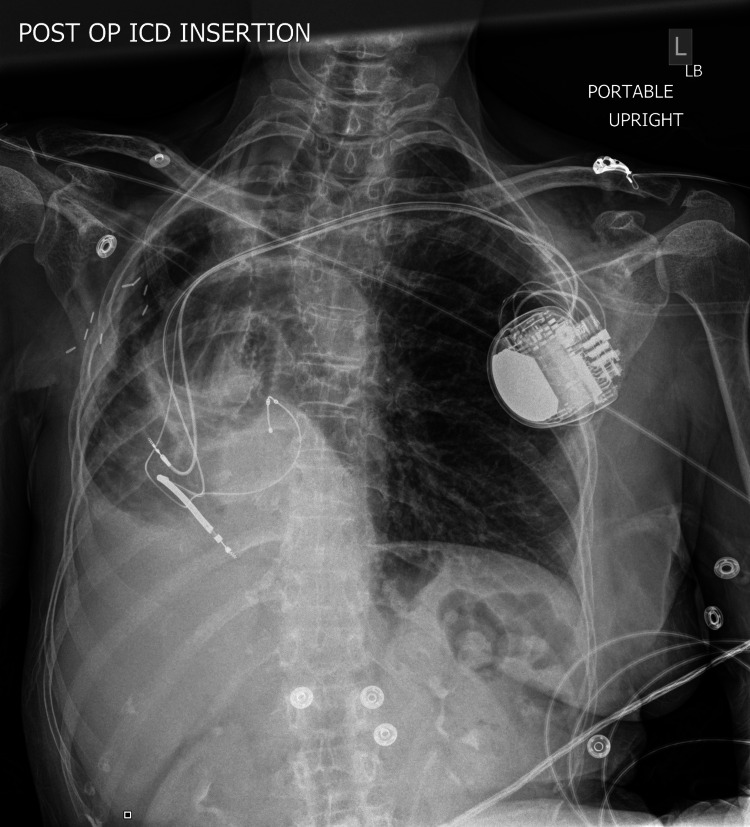
Chest X-ray (posteroanterior (PA) view) Post-ICD implantation leads and pseudo-dextrocardia are visible in the image. ICD: Implantable cardioverter-defibrillators.

**Figure 5:**
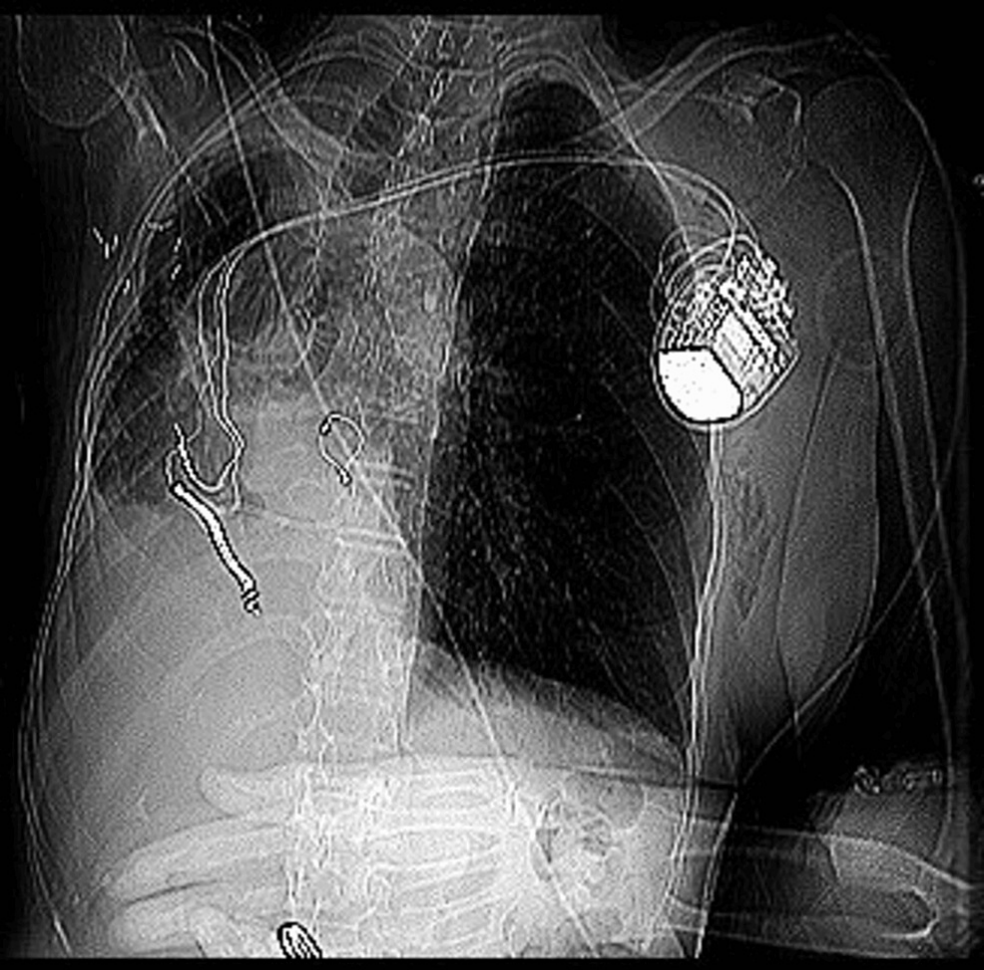
Post-intervention chest CT scan The image shows implantable cardioverter-defibrillator (ICD) leads and pseudo-dextrocardia.

The implantation of a defibrillator (Medtronic Claria MRI Quad CRTD, Minneapolis, USA) was performed using blunt dissection and electrocautery and a pacemaker pocket was created immediately anterior to the left pre-pectoral fascia. Using the modified Seldinger technique and fluoroscopic visualization, a micro-puncture access kit was utilized to get access to the axillary vein. A guidewire was passed into the right atrium and a coronary sinus long sheath was advanced over the wire. The guidewire was passed into the coronary sinus and the sheath was advanced over the wire. Contrast dye was injected to confirm placement in the coronary sinus. A balloon-tipped catheter was advanced through the coronary sinus sheath. An occlusive venogram was performed which revealed that the patient had a posterolateral branch. The balloon-tipped catheter was then removed. The right ventricular and right atrial leads were placed at the right ventricular apex and right atrial appendage, respectively. Cannulation of coronary sinus os was difficult due to the rotation and displacement of the heart. After multiple attempts, an angioplasty wire was successfully passed through the coronary sinus sheath into the distal point of the postero-lateral branch of the coronary sinus. A lead was passed over the wire and secured to the pre-pectoral fascia. All three leads were connected to the biventricular ICD generator (Figures 4-5). 

**Figure 6:**
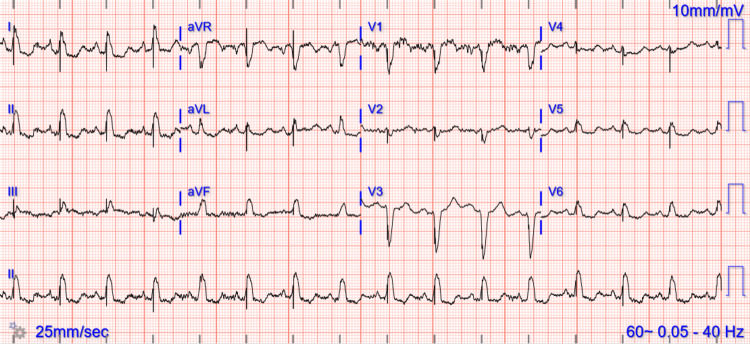
Post ICD (CRT-D) ECG ICD: Implantable cardioverter-defibrillators; CRT-D: Cardiac resynchronization therapy with a defibrillator; ECG: Electrocardiogram.

The patient was noted to have excellent sensing, threshold, and impedances (Table 1). Post-implantation ECG is shown in Figure 6. There were no procedural-related complications, and the patient was discharged the next day in stable condition.

**Table 1:** ICD Parameters ICD: Implantable cardioverter-defibrillators; LV: Left ventricular.

P-waves measured	1.4
R-waves measured:	6.5
Atrial Impedance	380
Right Ventricular Impedance	456
Left Ventricular Impedance	893
Shock impedance	149
Atrial Threshold	1.375 volts at 0.4 millisecond
Ventricular Threshold	0.75 volts at 0.4 milliseconds
Right Ventricular Threshold	0.75 volts at 0.4 milliseconds
Programmed Parameters	DDDR 60/130
Atrial Output: 3.5 volts	3.5 volts
Right Ventricular Output	3.5 volts at 0.4 milliseconds
Left Ventricular Output	3.5 volts at 1.0 millisecond
LV pacing vector	True bipolar

## Discussion

Radiation-induced lung injury (RILI) is a common complication following radiotherapy. Such a condition encompasses any lung toxicity induced by ionizing radiation. It presents acutely as radiation pneumonitis and chronically as radiotherapy-induced pulmonary fibrosis (RIPF). Incidences of RIPF range from 16-28% [3]. This case represents a severe form of RIPF, which resulted in a remarkable mediastinal shift.

RIPF presents clinically with a wide spectrum of symptoms and signs. The majority of patients are asymptomatic; however, symptomatic patients tend to present with dry cough, and exertional dyspnea [4]. A critical factor in RILI development is the dose of radiation given. Patients who receive doses of radiotherapy of more than 20 Gy are at higher risk of developing RILI [5]. Other important risk factors include the volume of the lung that has been irradiated and the technique used in radiotherapy.

There is one main strategy, and another potential one, to prevent RIPF development. Firstly, radiation-wise, limiting the dose of radiation to less than 20 Gy is associated with less risk of RILI development [6]. Also, proper radiotherapy technique is crucial to prevent the pathogenesis of RILI. Stereotactic body radiation therapy (SBRT), and proton beam therapy are the most effective techniques to prevent RILI with the latter achieving lower rates of RILI in less than 1% of patients [7-9]. SBRT is an external beam radiation therapy method that precisely delivers a high dose of radiation to an extracranial target within the body, using either a single dose or a small number of fractions [10]. Proton beam therapy is highly conformal radiotherapy where proton particles are used instead of the conventional X-ray to target tumors with higher radiation doses while halving total body radiation [11-14]. Lastly, using localized radiation techniques such as intensity-modulated radiotherapy (IMRT) and image-guided radiotherapy (IGRT) have resulted in minimal acute and chronic side effects compared with conventional therapies [15].

The other potential strategy encompasses potential medications that can attenuate RILI and slow (or prevent) the progression of RIPF. Pirfenidone, an antifibrotic agent, is a promising drug in slowing the progression of RIPF [16]. In murine models, pirfenidone was associated with attenuating RIPF and prolonging survival time in mice exposed to high-dose radiation [17]. Currently, a randomized clinical trial is underway to explore the potential benefits of pirfenidone in preventing RILI development in patients who underwent radiation for esophageal cancer [18]. The RELIEF trial studied progressively fibrotic interstitial lung disease (ILD) patients (other than idiopathic pulmonary fibrosis) and reached a conclusion that pirfenidone can attenuate the disease progression as evidenced by slowing the decline in forced vital capacity (FVC) [19].

Dextrocardia is yet another cause of heterotaxia, which presents with a right-sided heart, with an apical axis towards the right, and reversed aortic and vena cavae positioning. However, this patient has dextro-positioning of the heart with the apical axis to the left which is known as “pseudo-dextrocardia” [20,21]. Indeed, there was difficulty in cannulation of the coronary sinus ostium in this anatomical variant; however, cannulation was successful after multiple attempts. There are certain risks associated with ICD devices including infection, pneumothorax, coronary sinus perforation, and bleeding, benefits certainly outweigh the risks as ICD demonstrated improved long-term survival in such patient population [22]. There is an agreement between major professional societies’ guidelines in advocating the use of CRT for patients with QRS >150 ms and LBBB morphology as in the patient featured in this article. The present case demonstrates that ICD can be considered in patients with complex heart orientation. A similar case report revealed a successful ICD implantation in a patient with complex congenital heart disease, dextrocardia, and situs solitus [23]; however, studies with larger patient populations and long-term follow-up periods are required to generalize the safety and efficacy of ICD in severely displaced cardiac presentation as in this study.

## Conclusions

This case highlights the challenges encountered in the management of a geriatric patient with radiation-induced pulmonary fibrosis leading to significant cardiac displacement and rotation, a condition known as pseudo-dextrocardia. Despite the anatomical complexities, successful biventricular ICD implantation was achieved after persistent efforts. The case underscores the importance of adapting procedural techniques to accommodate unusual anatomical variations. Additionally, it underscores the potential benefits of ICD therapy in patients with complex heart orientations, providing a valuable option for those at risk of sudden cardiac death. While this case sheds light on a rare scenario, further research with larger patient cohorts and extended follow-up periods will be crucial in establishing the safety and efficacy of such interventions in similarly challenging anatomical presentations.
